# Review of epidemiological risk models for foot-and-mouth disease: Implications for prevention strategies with a focus on Africa

**DOI:** 10.1371/journal.pone.0208296

**Published:** 2018-12-13

**Authors:** Bachir Souley Kouato, Kris De Clercq, Emmanuel Abatih, Fabiana Dal Pozzo, Donald P. King, Eric Thys, Hamani Marichatou, Claude Saegerman

**Affiliations:** 1 Research Unit in Epidemiology and Risk Analysis Applied to Veterinary Sciences (UREAR-ULiège), Fundamental and Applied Research for Animals & Health (FARAH) Centre, Faculty of Veterinary Medicine, University of Liege, Liege, Belgium; 2 Institut National de la Recherche Agronomique du Niger (INRAN), Niamey, Niger; 3 Operational Directorate Viral Diseases, Unit Vesicular and Exotic Diseases, Veterinary and Agrochemical Research Centre (CODA-CERVA), Brussels, Belgium; 4 Department of Mathematics, Computer Sciences and Statistics, University of Gent, Krijgslaan Gent, Belgium; 5 The Pirbright Institute, Ash Road, Pirbright, Surrey, United Kingdom; 6 Department of Biomedical Sciences, Institute of Tropical Medicine, Antwerp, Belgium; 7 Université Abdou Moumouni de Niamey, Faculté d'Agronomie, Niamey, Niger; The University of Melbourne, AUSTRALIA

## Abstract

Foot-and-mouth disease (FMD) is a highly infectious transboundary disease that affects domestic and wild cloven-hoofed animal species. The aim of this review was to identify and critically assess some modelling techniques for FMD that are well supported by scientific evidence from the literature with a focus on their use in African countries where the disease remains enzootic. In particular, this study attempted to provide a synopsis of the relative strengths and weaknesses of these models and their relevance to FMD prevention policies. A literature search was conducted to identify quantitative and qualitative risk assessments for FMD, including studies that describe FMD risk factor modelling and spatiotemporal analysis. A description of retrieved papers and a critical assessment of the modelling methods, main findings and their limitations were performed. Different types of models have been used depending on the purpose of the study and the nature of available data. The most frequently identified factors associated with the risk of FMD occurrence were the movement (especially uncontrolled animal movement) and the mixing of animals around water and grazing points. Based on the qualitative and quantitative risk assessment studies, the critical pathway analysis showed that the overall risk of FMDV entering a given country is low. However, in some cases, this risk can be elevated, especially when illegal importation of meat and the movement of terrestrial livestock are involved. Depending on the approach used, these studies highlight shortcomings associated with the application of models and the lack of reliable data from endemic settings. Therefore, the development and application of specific models for use in FMD endemic countries including Africa is encouraged.

## Introduction

Foot-and-mouth disease (FMD) is a highly infectious transboundary disease that affects domestic and wild cloven-hoofed animal species. The disease has tremendous direct and indirect economic consequences resulting mainly from constraints in international trade in animals and animal products originating from infected countries, as well as costs associated with controlling disease outbreaks [1,2]. However, FMD consequences are not the same throughout the world [3] as the impacts of the disease vary markedly between FMD endemic and FMD non-endemic countries, developed and developing countries, and also within many developing countries [4]. The etiological agent of FMD is a small, non-enveloped, positive-sense, single stranded RNA (8.4 kb in length) virus belonging to the genus *Aphthovirus* of the family *Picornaviridae* called foot-and-mouth disease virus (FMDV). The primary mode of transmission of FMDV is via direct contact from infected to susceptible animals [5]. The virus can also be spread mechanically by contaminated organic debris, fomites or personnel and materials from infected farms that may carry the virus to susceptible animals in another farm [6–8]. Furthermore, FMDV transmission can also be airborne, a mechanism by which virus exhaled into the air by infected animals can be spread over long distances depending on the wind speed and direction [9,10]. Additionally, FMDV can be transmitted locally between livestock housing of susceptible animals when there is no clear linkage other than geographical proximity [11]. This occurred in 2001 in the UK after the introduction of movement restrictions following the first FMD outbreaks. In these circumstances, the spread was limited to a 3 km distance from an infected premise [12, 13], however long distance spread continued to occur even at a reduced level [13]. The rapid spread of FMDV highlights the need for measures to effectively prevent and/or control the disease. Development of control policies for different scenarios requires a deep understanding of FMD epidemiology that can be supported by accurate and relevant epidemiological models [14].

There are several reviews of FMD models in the literature [15–18], but it is perhaps not surprising that many of these are focussed on the large-scale UK epidemic in 2001, which in many aspects was responsible for pioneering a step-change improvement in these tools. Consequently, most of the models related to FMD transmission were designed for use in countries where the disease is not endemic, where control measures are implemented to contain a single virus incursion into a naive population and recover FMD-free status as quickly as possible. However, in endemic settings, different factors play a critical role in virus circulation and require consideration such as waning of natural immunity or vaccine-induced immunity, and frequent disease re-introduction as well as the potential involvement of wildlife reservoirs [19]. Consequently, it is difficult or even wrong to extrapolate the experience in one country to another one as farming practices, farm density, farm size, and contact patterns may differ [20]. In contrast to most developed regions where FMD has been eradicated, the disease is still endemic in most of Asia, Africa and parts of South America [21]. In many of these endemic settings, there is no efficient control plan as FMD risk factors are poorly understood, and most of the parameters required for models are not well understood. These endemic areas constitute a real and permanent threat for FMD free countries through numerous transmission pathways. Considering the need to mitigate this potential event of FMDV entry from endemic to non-endemic FMD countries, the implementation of FMD risk assessment in endemic areas such as Africa is warranted. However, the most relevant question to be addressed is whether well-formulated and tailored FMD models exist for determining appropriate control policies in endemic countries.

A range of analytical techniques exists with specific uses ranging from FMD outbreak response planning (e.g. risk analyses, simulation modelling studies, mathematical modelling studies) to understanding which farms are at risk of infection, once an incursion has occurred (regression models) (**Table 1**). Therefore the aim of this review was to systematically collect information on studies related to some risk models for FMD that are well supported by scientific evidence from the literature. This review will specifically focus on the use of modelling in an FMD endemic context such as Sub-Saharan Africa (SSA) to inform recommendations on critical FMD prevention and control options.

**Table 1:** Categories and objectives of main modelling techniques.

Category	Model	Objective
Regression models	Logistic regression	Quantify the association between a set of explanatory variables and either the presence or absence of an FMD outbreak at a given location
	Poisson regression	How large an outbreak will be?
Mathematical models	Susceptible-exposed-infected-recovered model	Understand the likely effect of different control strategies (e.g. restriction of movement, use of vaccination
Simulation models*	NAADSIM [123] InterSpread Plus [11] AusSpread[124]	Provide detailed information for response planning (e.g. explicit estimation of human resource requirements)
Risk analyses	Risk of release	Used to estimate when and where an FMD incursion might occur
	Risk of exposure	Once an FMD incursion has occurred, what is the likelihood that other farm premises will be exposed?

* Simulation models are used in a similar way to mathematical models, but they tend to have greater flexibility allowing the effectiveness of control measures to vary geographically and over time.

## Materials and methods

### Systematic review

#### Literature search process

Relevant published articles were searched based on the PRISMA (Preferred Reporting Items for Systematic reviews and Meta-Analyses) method [22].The search was conducted through online search engines, particularly in PubMed (www.ncbi.nlm.nih.gov/pubmed) and Scopus (www.scopus.com) using different combinations of seven keywords. These keywords were: (a) "Foot-and-Mouth Disease", (b) "Modelling", (c) "Risk assessment", (d) "Risk factors", (e) "Spatiotemporal", (f) "Transmission" and (g) "Spread". The search was restricted to articles written in English or in French, with an available abstract and published between January 1997 and December 2016. Two screening steps were applied based on defined inclusion and exclusion criteria **(Table 2)**. The first step was applied to the titles and abstracts to select potentially relevant papers, while the second stage of screening was applied on the full text. Additionally, some other documents were identified from the references of included articles and were added to the present review.

**Table 2:** Inclusion and exclusion criteria for peer-reviewed studies included in this review.

Inclusion criteria	Exclusion criteria
• Studies should be original articles published in a peer-reviewed journal during the last 20 years (from 1997 to 2016)	• Studies related to another pathogenic agent (such as enteroviruses) instead of FMDV
• Studies should focus on different spatial and spatiotemporal models to estimate the risk of occurrence or transmission of FMD	• Studies reporting the use of biological models rather than statistical or mathematical models
• Studies describing quantitative and/or qualitative risk modelling of FMD	• Articles describing models of the transmission dynamics of FMDV spread through populations, or compartmental models
• Studies reporting patterns of different epidemiological outbreaks in terms of FMDV spatiotemporal distribution	• Modelling studies reporting the exploration of either different strategies or resource requirements in hypothetical outbreaks (simulation models)
• Retrospective analysis of historical outbreaks data with the purpose to highlight FMD risk factors	• Articles describing only the modelling of economic impact of FMD • Studies carried out to assess laboratory tests or the performance of surveillance systems (sensitivity and specificity) • Experimental studies related to factors associated with secretion and excretion of FMDV • Modelling studies that did not explicitly discuss FMDV transmission and risk factors for its spread

#### Data collection and analysis

To be included in the analysis of this review, the following had to be available for the retrieved papers: (1) the country of interest, (2) the type and features of the model, (3) the mode of transmission discussed in the study, (4) the assessment process, (5) details of the main risk factors involved in the transmission, (6) and if any details of the practical implications arising from the study. The extracted data were compiled in an Excel datasheet and subsequently a descriptive analysis was performed to provide state-of-the-art insights on FMD epidemic models and risk analysis.

## Results

**Fig 1:**
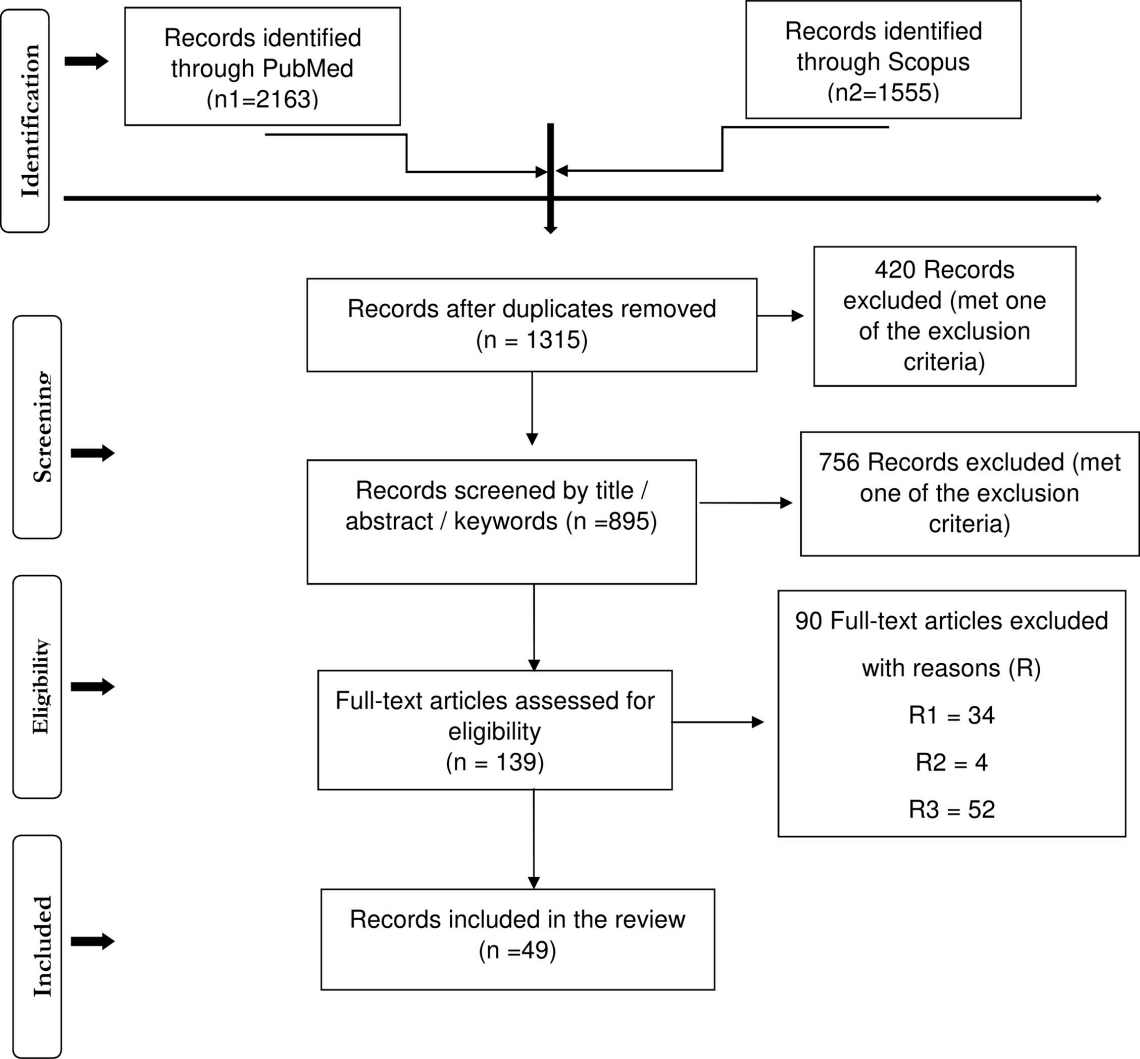
The Preferred Reporting Items for Systematic Reviews and Meta-Analyses (PRISMA) Flow Diagram. R (reason) 1: UK FMD 2001 epidemic models; R2: Japan 2010 FMD epidemic models; R3: Other simulated epidemic models.

The literature search yielded 3718 records through the two databases (PubMed and Scopus). After removing duplicates, 1315 unique publications were identified as potentially relevant references and were screened using titles, abstracts and keywords. Out of these screened articles, 139 full texts were assessed for eligibility. A total of 124 references were selected and presented in this review, including 75 additional articles retrieved after screening the reference lists of the eligible papers given that the 49 retrieved published papers met at least one of the inclusion criteria. The flow diagram in **Fig 1** shows the search process. The PRISMA check list, the search strategies, and the results for the consulted databases are provided in **S1 and S2 Tables** respectively.

### Study selection

Based on the results of the literature search process, the selected articles in this systematic review were categorized into two types: (1) modelling FMD risk factors and spatiotemporal analysis, (2) FMD risk assessment models, further subdivided into two components (quantitative and qualitative). Hence, out of the 49 included articles, 14 described quantitative risk models, 5 were related to qualitative risk assessments while 30 reported results of spatiotemporal or risk factor analysis (**S3 Table**).

**Fig 2:**
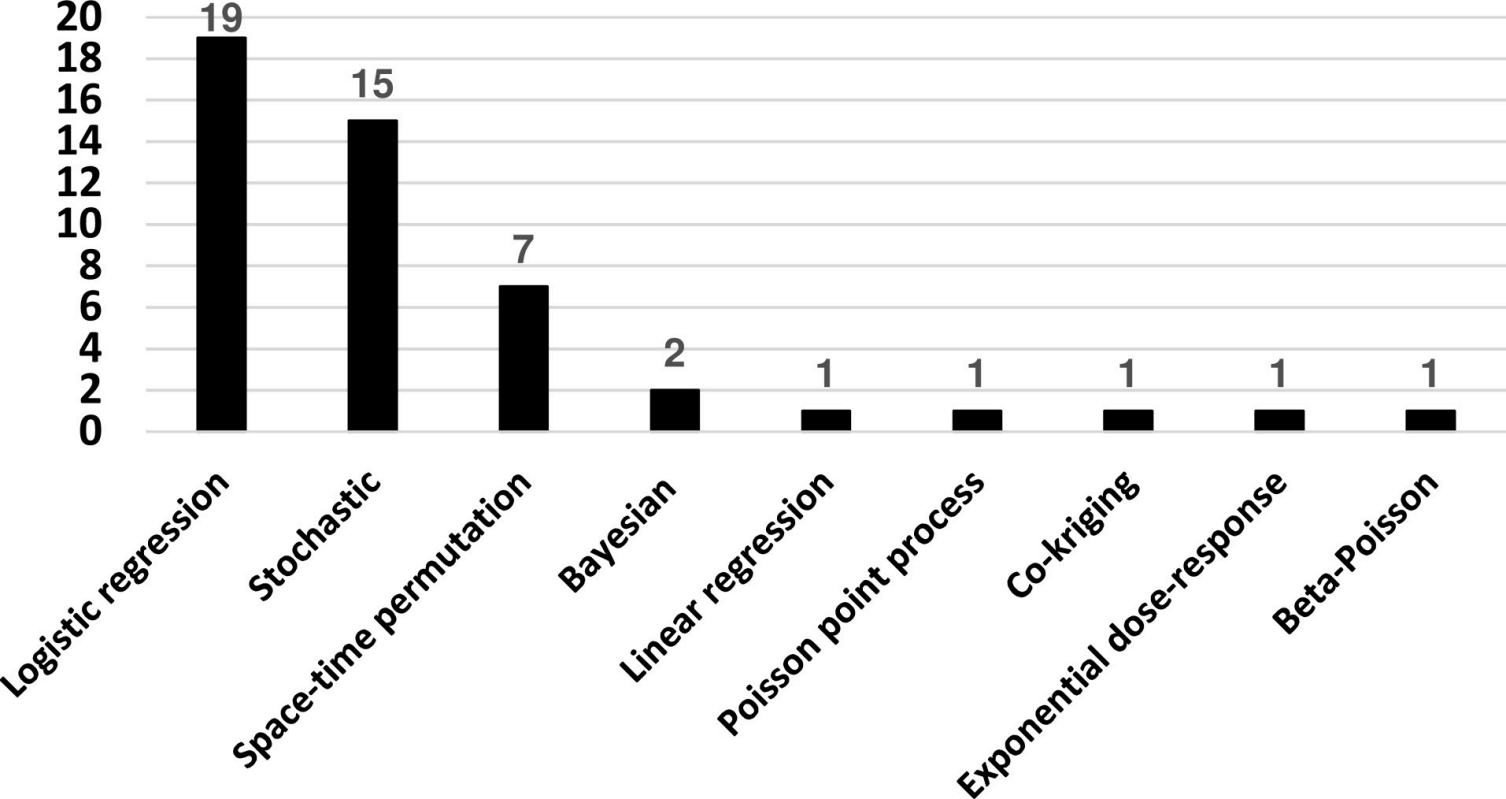
Frequency of type of models among the studies included in this review.

The chronology of publication of the included articles indicated that the attention to risk modelling is relatively recent. Although the use of a type of mathematical or statistical models depends on the purpose of the study and the nature of the data, logistic regression and stochastic models were the most frequently used in the modelling studies included in this review (**Fig 2**). Regarding the geographical origin of articles related to risk modelling, it is not surprising that many studies were implemented in developed countries, which are free of the disease. However, a significant number of spatiotemporal and risk factor analysis studies were performed in endemic countries or regions such as Sub-Saharan Africa.

### General overview of modelling techniques used for FMD

Although mathematical modelling had been used as a tool in veterinary epidemiology for many years before 2001, the FMD epidemic in UK that year was the first situation in which techniques were developed in the ‘heat’ of an epidemic and used to guide control policy [23].

**Fig 3:**
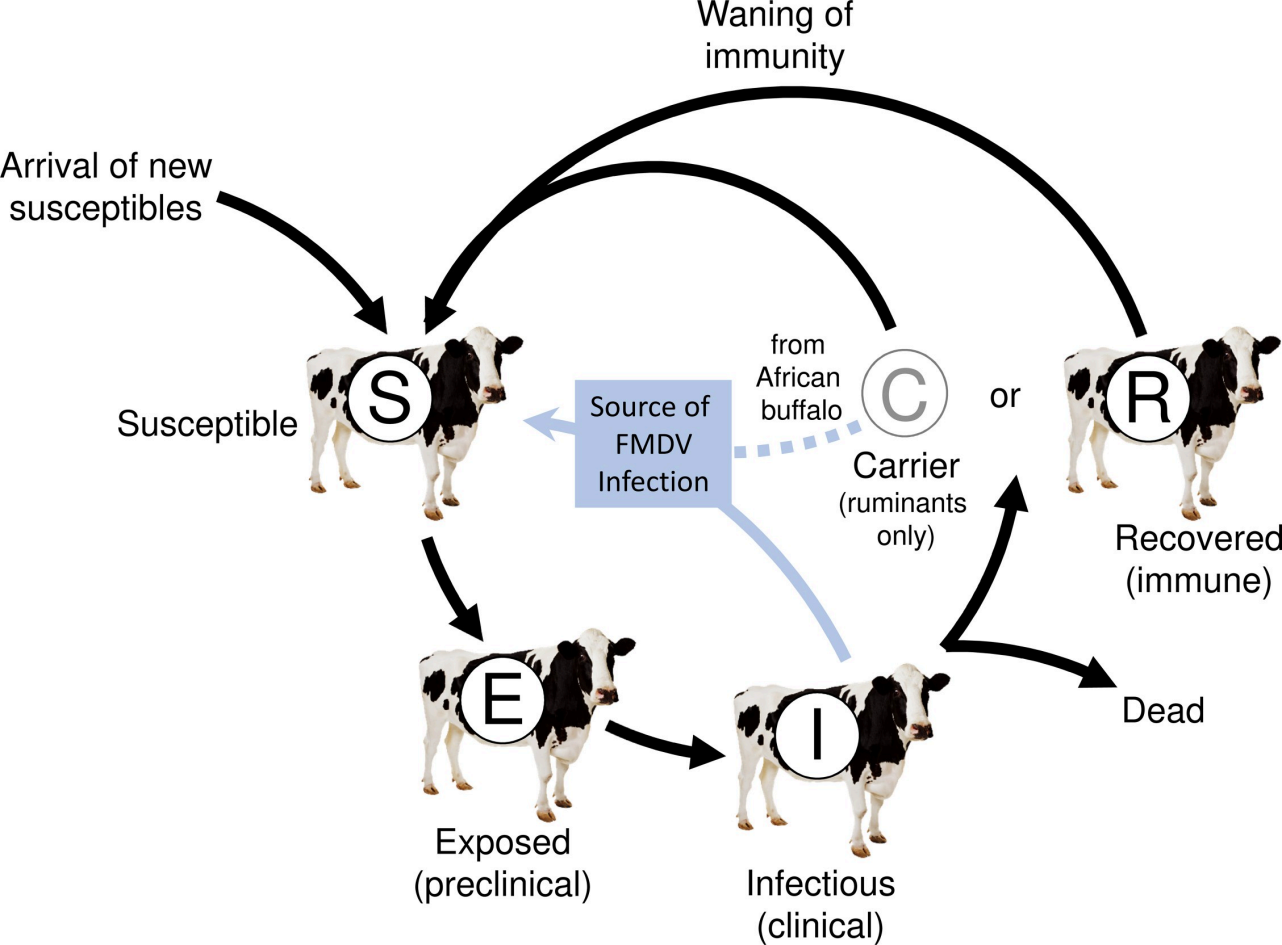
Schematic representation of an epidemic model (Source: Adapted from Kostova et al., [122]).

These techniques provide a representation of the transmission dynamics of infectious diseases among animals, and/or among groups of animals in time and/or space [24,25] (**Fig 3**). Indeed, the modelling approach of infectious disease is to divide the host population into different compartments denoted S, E, I, R representing respectively Susceptible, Exposed (infected but not yet infectious), Infected and infectious and Recovered (or removed) animals or premises. The dynamics of infection are then represented by the movement of hosts from one compartment to another. Such a model is also referred to as a SLIR model, with S, L, I, R representing respectively Susceptible, Latent, Infected and infectious and Recovered (or removed) animals or premises. If vaccination is involved, there may also be a compartment denoted V representing vaccinated animals or premises [25]. Simulation models are used in a similar way to mathematical models, but they tend to have greater flexibility allowing the effectiveness of control measures to vary geographically and over time. Indeed, all of these techniques can contribute to contingency and planning through exploration of the resource requirements of different strategies in hypothetical FMD epidemics [26, 27].

Although, there is no agreed classification system for models. Several authors have focused on different aspects of models, which may distinguish them from each other. According to the treatment of probability, variability, and uncertainty, models can be stochastic or deterministic. Models, which assign averages or most likely values to all parameters and model the average or most likely outcome of probability events, are named ‘deterministic’ models. They produce a single output or result for each set of input values or scenario [23]. For example, deterministic models were used by Ferguson et al. [28,29] for the FMDV epidemic in the UK in 2001. Models, which included the effect of probability and variability, are termed ‘stochastic’. As parameter values within the model can vary and the occurrence of chance events is random, stochastic models must be run repeatedly to produce a range of outcomes from the same input scenario. Such models were used by Keeling et al., [13] also in the 2001 FMD epidemic in the UK.

In analytical epidemiology including FMD risk factors analysis, regression modelling is one of the most important statistical techniques used to investigate the effect of one or several explanatory variables (e.g., exposures, risk factors) on a response variable such as mortality rate or disease occurrence [30]. Regression models are useful because they allow analysts to identify when and where outbreaks are likely to occur (logistic regression) or how large an outbreak will be (Poisson regression). Hence, depending on the nature of the data, three regression models were mainly used in epidemiological studies: logistic regression, Poisson regression and alternatively negative binomial regression.

1. Logistic regression models are used to measure the association between a set of explanatory variables and either the presence or absence of an FMD outbreak at a given location [31].
2. Poisson regression models are used to analyse count data as a function of a set of predictor variables. These methods are suitable to quantify the number of infected places identified over a given time frame. However, these models have many applications, not only to the analysis of counts of events, but also in the context of models for contingency tables and the analysis of survival data [32]. Poisson regression assumes the response variable Y has a Poisson distribution and a logarithmic link function. Indeed, it assumes that the logarithm of its expected value can be modelled by a linear combination of unknown parameters. A Poisson regression model is sometimes known as a log-linear model, especially when used to model contingency tables. In this context, Poisson regression models are used to estimate incidence risk or incidence rates adjusting for important factors or confounders.
3. when over dispersion of data is a problem in the Poisson regression, an alternative is the use of a negative binomial regression [33, 34]. This alternative distribution may be used to determine the relationship between incidence risk/rate of FMD outbreaks by (e.g.) space, province or district), time (year or month), vaccine coverage or density of population like the number of bovines per province or district.

Spatial effects may play important roles in the spread of diseases including FMD. The scan statistic is commonly used to test if a one-dimensional point process is purely random or if any clusters can be detected [35]. In this respect, the spatial scan statistic has been one of the most commonly used techniques to address spatial clustering of animal diseases, FMD included [35–38]. Some of the advantages of the technique include the ability to identify specific high-risk areas, as well as to quantify the risk [36,39]. This type of model makes minimal assumptions about the time, geographical location, or size of the outbreak, and it adjusts for natural purely spatial and purely temporal variation. In this technique, a hypothetical spatiotemporal cylinder is centred at the geospatial coordinates of each location where outbreaks had been reported. The base and the height of the cylinder represented, respectively, the spatial and temporal dimensions for each potential cluster of outbreaks. The base and height of the cylinder were let to vary up to a maximum size equivalent to the inclusion of 50% of the reported outbreaks [40]. A condition of the time–space permutation model of the scan statistic is that the numbers of cases within the spatiotemporal cylinder are Poisson distributed. This condition can be assumed when the number of cases per day (cd) and the number of cases per area (ca) are small, compared with the overall number of cases.

### Modelling FMD risk factors and spatiotemporal analysis

Out of the 30 studies reporting spatiotemporal and risk factor modelling of FMD, 20 were designed as retrospective studies using mostly historical data and were often associated with survey results based on questionnaires [36–39, 41–56]. Among this type of selected studies, 4 were designed as case-control [49–52], 4 others were conducted as cross-sectional or seroprevalence studies [57–60] and 2 modelling studies were performed based only on questionnaires data [61,62]. A table is provided in the appendix (**Part A of S3 Table**), to summarise the key features of the different FMD modelling techniques used in studies included in this systematic review.

Despite the geographical diversity of the studies, there were indeed some common risk factors. The most frequently reported factor was the animal movement *sensu lato* (**Table 3**). The uncontrolled animal movement leads to other risk factors such as mixing of animals around water and grazing points, a risk factor that is widely identified in Africa, undoubtedly linked to the farming and transhumance practices. However, there are some specific risk factors like the contact between wild animals and domestic animals which are more relevant in Africa [52,62–63], and animal density which is predominant in Europe [44,45,54]. The other identified risk factors such as the seasonal pattern of occurrence of FMD outbreaks [41,43,64] or the factor of susceptibility related to the age of animals [48,58,65] were less frequently reported in the selected studies.

**Table 3:** Main risk factors identified through selected modelling studies and presented in this review (sorted by decreasing order of occurrence).

Identified main risk factors	Country of interest	Reference
Animal movement	Tanzania, Uganda, Cameroon, Togo/West Africa, Turkey, Zambia, Ethiopia, Japan, Pakistan,	[39,42–43,47,49,51,53,54,60,62,63,88]
Animal trade	Cameroon, Togo/West Africa, Iran, Ethiopia, Pakistan, Zambia, UK	[39,51,54,55,65,62,88]
High animal density	Japan, Ethiopia, Iran, UK, Turkey	[36,44,45,47,50,51,56]
Mixing of herds (around water points and on pastures)	Uganda, Cameroon, Bhutan, Nigeria, Zambia, Ethiopia	[43,49,59, 61,63,88]
Contacts between domestic animals and wildlife	Tanzania, Nepal, Zambia, South Africa	[42,53,54,62,64, 88]
Human activities and / or lack of compliance with biosecurity measures	Tanzania, Nepal, UK, Japan	[38,42,46,57,60]
Seasonal pattern of occurrence of FMD outbreaks	Middle East, Uganda, South Africa	[41,43,64]
Young animals identified as being most susceptible to infection	Israel, Iran, Bolivia	[48,58,65]
Lack of early screening/detection	UK	[52,55]
Shorter distances to the nearest infectious source	UK	[37,45]

### FMD risk analysis models

There are two main approaches to risk analysis: qualitative and “quantitative. In a qualitative risk analysis, the risk level is appreciated in qualitative terms; like, for example, “the risk of introduction is “negligible”. In a quantitative analysis, the risk is appreciated in quantitative terms e.g. by risk rates, usually as a probability. Additionally, there is broad agreement concerning the definition of risk analysis defined as *"A process consisting of three components*: *risk assessment*, *risk management and risk communication”* and, for risk assessment, defined as *“A scientifically based process consisting of the following steps*: *(i) hazard identification*, *(ii) hazard characterization*, *(iii) exposure assessment*, *and (iv) risk characterization"* [66].

### Quantitative risk assessment model

In this review, 14 articles presenting a quantitative analysis of FMD risk were selected. In quantitative risk analysis, Monte Carlo simulation is usually used to assimilate the probability components of the import scenario. Several software programmes have been developed within a spreadsheet environment for Monte Carlo simulation. The uncertainty associated with an input and its known variability was modelled as a probability distribution. Although the electronic search yielded only few articles, published in recent years, risk analysis has been earlier applied in the field of animal health, particularly in food safety (microbiological risk assessment) and import risk analysis (IRA), including number of studies on FMD risk assessment. Indeed, most of the studies reported risks related to the importation of potentially contaminated animal products (milk or meat) [67–69] or live animals [70–72]. Some studies were related to the risks associated with movement of either people or animal products possibly infected with FMDV [73,74]. Most reviewed IRAs originated from FMD free countries, mainly in Europe and USA [68,70,71]. Only one included published study on quantitative risk assessment was performed in an FMD endemic country namely Zimbabwe [75]. Through these quantitative risk assessment studies, the critical pathway analysis showed that the risk of FMDV entering a country is overall low [8,67,70,73–77]. However, depending on the research question and model assumptions, some risks could be considered as relatively high depending on their nature, i.e. the illegal importation of meat and the terrestrial movement of livestock [68,69,78–79] (listed in **Part B of S3 Table**). The reviews performed by Garland & De Clercq [80] and by Potier [81] related to the risk assessment approach were not included in the analysis of this review, based on the exclusion criteria. However, important insight has been provided by these reviews, for instance, Garland & De Clercq [80] reported a comprehensive review of risk assessment related to vaccinated animal import. It was demonstrated through this review that the risk from products derived from vaccinated animals is very low when risk mitigation measures are correctly applied.

### Qualitative risk assessment models

Based on the method of data extraction used in this review, the key findings of the included articles related to FMD qualitative risk assessment (n = 5) were summarized in a narrative description of each study. Taking into account the design of these studies, an exception was made to include some published reviews with respect to the defined time frame of publication which is between 1997 and 2016. In general, FMD qualitative risk assessment was based on the OIE assessment framework, using available data from published sources and various unpublished sources [82–84]. As mentioned above, the main application of risk analysis in the animal health field has been directed to import risk analysis, which is the assessment of disease risks associated with international trade in animals and their products. This is illustrated by the research question of some included articles, which served as basis for the qualitative assessment of risk [83–86]. However, for both quantitative and qualitative risk analysis, the fields of application of these assessment methods were extensive and diverse [17,82,87]. Notwithstanding, these studies revealed some risks that ranged from negligible to moderate (**Part C of S3 Table**). Based on these qualitative assessments the authors proposed useful or important recommendations for the prevention and control of FMD.

## Discussion

### FMD risk factors and spatiotemporal distribution modelling

This review demonstrated that in the field of FMD epidemiology, several studies have been performed with the aim to show that a given risk factor contributes to the occurrence and/or transmission of the disease. However, it is likely that some identified risk factors are not causative and merely reflect increased risk via association with other risk factors. To this regard, logistic regression is a commonly used analytical technique for FMD risk factors analysis [44–49,51–53,57–61,65–66,88]. One of the advantages of such an approach is that specific risk factors can be identified and their impact quantified, managed or controlled [88]. On the other hand, this review showed the importance of using spatiotemporal models like the space-time scan statistic permutation model [38,42,46,54]. Indeed, assessing the spatiotemporal clustering of FMD prevalence or incidence appears to be a useful method for identifying geographical regions and periods of time in which the disease is more likely to occur. Hence, in the identified significant clusters, further FMD investigation should be implemented to identify predictors for outbreaks and epidemics to improve the effectiveness of preventive plans in reducing the occurrence of disease outbreaks [39]. In addition, better appreciation of spatiotemporal aspects of an epidemic means better practical decision making (e.g. pre-emptive culling radius, surveillance period, and resources required and appropriate effort to trace the spread of infection from infected premises) [57]. In our point of view, this is greatly needed, specifically in the context of endemic countries in SSA with a broad common pastoral space but mostly with limited financial and logistical resources.

The selected papers highlighted several factors that contribute significantly to the occurrence of FMD outbreaks. Even though these studies were carried out in different geographical areas, the predominant risk factor of FMD remains the uncontrolled animal movements (e.g. [89]). Other risk factors, such as mixing animals around water points, on pastures and in livestock markets were also elucidated. Nevertheless, it should be noted that the magnitude of these risk factors, most likely related to the farming system, do not have a similar impact on the prevalence or incidence of the disease as well as on the control measures to be implemented. For example, during the UK FMD epizootic in 2001, in addition to the policy of slaughtering animals on infected farms, further control measures were initiated, including a ban on all animal movements, the closure of markets, and the restricted public use of footpaths across agricultural land [29]. In contrast, in endemic countries with a huge epidemiological complexity and considering the livestock production system such as the transhumance or nomadic system, the application of the prevention and control options mentioned above would be unrealistic. Indeed, the context is so far different from that which prevails in several SSA countries where the animal husbandry system includes a seasonal cyclical movement, and where large herds must migrate over long distances in search of grass and water, within the country of origin or by crossing over the border to neighbouring countries (transboundary transhumance). This favours the contact between infected and healthy animals and between potentially infected wildlife and domestic animals and as a result induces a significant risk of disease spread, FMD included [43,62,88,90–91]. However, there are specific risk factors for certain regions such as the presence of wildlife which plays an important role in the maintenance of FMDV of SAT serotypes in Africa [92–95]. Some other studies identified risk factors including international livestock trade [40,89] and transboundary movements of animals, and stressed the absolute necessity for an integrated control at country, regional or continental level [96–98]. This could be based, for example, on coordinated vaccination programs against FMDV serotypes circulating within a region.

Even though there are numerous epidemiological modelling studies that have assessed risk factors and spatiotemporal distribution of FMD occurrence [23, 44, 50] most of them are related to particular epidemic episodes, mainly the 2001 FMD epidemic in Europe and, as a result, their findings cannot be extrapolated to all situations [42]. Indeed, these studies are useful but also some limitations and should be cautiously considered before implementation in Africa as not only their output but also the modelling methods used need to be translated onto local field conditions and considering available data. Although the technical development is identical, the application of models can and should vary based on the purpose of the research. In addition, some of the limitations of the risk factors analysis and of the spatiotemporal distribution could be related to the applied model type [99,100].

For example, in logistic regression analysis, large sample sizes are required to provide sufficient number of positive cases for proper estimation [58]. In addition, the explanatory variable should not be highly correlated with another variable because this could induce problems of estimation [89,99]. For illustration, from the articles describing the use of logistic regression, we have extracted and recorded in Table 4, data that highlights some limitations of logistic regression. For example, statistical power calculations were seldom reported in the included articles. On the other hand, the association between considered variables as well as the justification of the sample size are reported in these articles.

**Table 4:** FMD risk factors studies reporting use of logistic regression model.

	Number of included articles using logistic regression model	References
Justification of the sample size	10	[48,50–52,58, 61,64–65, 88]
Calculation of statistical power	1	[47]
Existence of correlation between discussed variables	10	[44–45,47,52,58,60, 63–65, 88]

The permutation model was also extensively used by some authors [41,36,54]. Nevertheless, it has a disadvantage due to the shape of the clusters constrained by the cylindrical shape (with a circular base) of the window used to scan the studied area. This could lead to a serious constraint when the geographical extension of the detected clusters is large [40]. The method detects only outbreaks that start locally, not those that occur more or less simultaneously in the whole surveillance area.

Another example of limitation due to the applied model is given by Perez *et al*., [39]. Indeed, these authors have used the co-kigring model to estimate the spatial risk of FMD in Pakistan. The co-kriging model uses information on covariates that are assumed to be associated with the outcome and to be known throughout the study area. Consequently, the findings of this type of study are formulated from a model that is based on a probabilistic interpolation method, which does not consider the variability of data resulting from various reporting systems [39].

The limitations of models in relation to the used data will be further discussed in the next section devoted to qualitative and quantitative FMD risk modelling. However, the limitations due to the use of questionnaires should be mentioned. Indeed, some authors presented a possible reporting bias when using data recorded by questionnaire rather than by using a prospective collection of objective data [51,59,60]. Using questionnaires, a confounding effect among variables has been reported [49,88]. For instance, Bronsvoort et al. [88], mentioned the existence of many variables relating to cattle density and management, the quality of veterinary services and other socio-economic factors, as well as possible ecological factors that could vary across the study area, which the questionnaire did not measure and will need further investigation with a much larger study. This pointed out the weakness of questionnaire-based studies, which is the potential for recall bias and the lack of possibility to validate questionnaire responses [88].

Likewise, the analysis of risk factors based on seroprevalence studies can present limitations related to the low sensitivity and specificity of the applied serological test [63–65].

### FMD risk assessment models

Despite the relatively few articles reporting risk assessment models (n = 19) collected for this review, it was observed that, in developed FMD free countries, FMD risk assessment modelling was performed, with the aim to estimate the risk of introduction of FMDV via several pathways including importation of animals or animal products [101–104]. Irrespective of the differences between the two approaches (quantitative *versus* qualitative), the decision-makers gained a thorough understanding of the FMD risk through risk assessment which resulted in sensible and realistic recommendations. If implemented, these recommendations can lead to a sustainable strengthening of capacities to prevent, control and even to eradicate FMD [18,87,105].

Given the risks estimated by the two assessment methods, the risk of introduction ranged overall from low to high. The interpretation of these results must be made cautiously. Indeed, the low level of an estimated risk is very different from the absence of the risk. Some authors explicitly reported the low level of risk in relation to the deficiency of available data to make their models more useful [8,70,71,74,75], although in some models, some values of parameters were either assumed based on expert opinion [68,71,73,76,77] or determined from experimental studies [67]. According to some authors, livestock movements do not represent a risk because the importation of susceptible live animals into FMD-free countries from countries that are not FMD-free is prohibited [85,86].

Depending on the used approach, the selected studies have also some shortcomings that can be ascribed to the risk assessment methodology. As noted above, qualitative risk assessments express risks in relative qualitative terms and often involve the aggregation of expert opinions. A comprehensive collection of data combined with expert opinion, was first undertaken by the European Commission for the Control of Foot and Mouth Disease (EuFMD), but thereafter extended and reviewed by the working group on FMD risk coordinated by the European Food Safety Authority (EFSA). This was done to assess the risk of FMDV entering through a pathway that could lead to its eventual release in the European Union from FMD risk regions such as Africa, Asia and South America [105]. To this regard, the methodology for qualitative risk assessment must be rigorous to ensure that the true risk, and not the false risk perception, is assessed as most likely, any decision can lead to a major animal health and economic impact [106]. Risk assessment can be also quantitative, i.e. providing a numeric estimate of the probability of risk and the magnitude of the consequences. Furthermore, quantitative risk assessment allows to model uncertainty and accordingly to undertake sensitivity analysis to determine the relative importance of variation in different inputs on the output(s) [67,68,70,73,74,76,77,79]. However, quantitative risk analysis may be too complex to carry out as they require more time, resources and accurate data. Indeed, a major and common problem for modelling is the lack of reliability and accuracy in recorded data [36, 41–43,51,54,55,58,63–65]. Similarly, it should be emphasized that several FMD endemic countries with substantial animal populations provide no information on FMD outbreaks or provide data that are considered to reflect a significant under-reporting of the true situation [105,107]. A distinguishing feature of the outbreak of FMD that occurred in the UK in 2001 is that detailed and factually correct data were collected throughout the epidemic. This data set has proven to be of enormous value for informing mathematical and simulation modelling studies [13,15,28, 29,108]. In a recently published review, Pomeroy *et al*., [109] elegantly demonstrated the crucial importance of data availability and accessibility for model implementation. Similarly, Hyeyoung *et al*., [110] demonstrated the unavoidable prerequisites of good-quality data to perform modelling. Indeed, they have more recently showed by simulation modeling the impact of the movements of mobile pastoralists on FMDV transmission in a transhumance system in the Far North Region of Cameroon. However, according to the authors a comprehensive explanation of the endemic feature of FMD in the concerned study area must include other factors such as the roles of sedentary and international transboundary herds and possible FMD carriers. Moreover, whatever the modelling approach (quantitative or qualitative), the uncertainty of each step of the model should be clearly underlined and reported to decision-makers.

Apart from the limitations related to the types of models and the quality of data used, some weaknesses of this review should also be noted. Some limitations could essentially be related to the search methodology applied. The time criteria as well as the Boolean operators used may have caused us to inadvertently miss pertinent research articles. For example, the use of the term “model” instead of “prediction” or “simulation” could probably result to miss certain published articles, which do not include these in their titles, abstracts and/or keywords one of these keywords. But, the Boolean operator “AND” was used between the two keywords “Foot-and-Mouth Disease" and "Epidemiology" to avoid this and typically this could encompass all epidemiological studies of FMD. Moreover, it excluded the epidemic (real or simulated) models, especially those based on UK FMD 2001 models and similar models. The heterogeneity of the selected studies, mainly in relation to the used assumption and parameters, was a major constraint for data extraction and accordingly it precluded a more extensive quantitative comparison between studies. In addition, risk factor analysis through seroprevalence studies could have some deficiencies because of the sensitivity and specificity of the diagnostic tests used [2, 59,63–65]. Consequently, this fact has unfortunately not enabled the ranking of the identified risk and the associated contributing factors.

One of the strengths of this review is to identify some FMD occurrence risk factors either at farm-level or animal-level. This subsequently may allow the proposition of some basic recommendations for preventive measures of FMD. First, it should be noted that the control measures depend largely on the epidemiological status of a given country or region, the livestock production system, but notably also on the available financial resources. For example, in developed countries, in case of an FMD outbreak, a recommended policy is to strictly implement stamping out (or pre-emptive culling when the risk of transmission or spread is present). Although the economic impacts are very high, these costs are usually covered by national compensation schemes that remove many of the objections to the application of these measures for the effective control of the epidemic. On the contrary, in developing countries, with most of them being FMD endemic, this option cannot reasonably be considered for many reasons including the financial losses to rural communities. Hence, forr the principal risk factor (animal movement) and other factors resulting from the movement (as mixing herds around water points and on pastures), the recommended control measure is the prohibition or restriction of movements during FMD outbreaks as much as possible. Considering that transhumance or nomadism system are dominant in some African regions like SSA, vaccination of animals before going on transhumance could effectively reduce the incidence of the disease. However, for implementing this measure, there is an ultimate need of an updated knowledge of FMDV serotypes circulating in the region. Indeed, the combined use of vaccination of animals every 6 months with improved methods for sero-surveys to monitor viral activity could be decisive to overcome the concerns that vaccination would hide infection [111]. For animal trade at local or national level, the application of quarantine measures should be strictly applied. In case of FMD clusters with a well-known seasonal pattern of occurrence of the disease, selective vaccination campaigns, surveillance activities and control of movements before and during the season at higher risk could be appropriate. Some studies reported that in detected FMD clusters, young animals are the most susceptible to FMD infection. Therefore, increasing the frequency of vaccination among herds followed by the intensification of surveillance activities (where young calves are abundant, surveillance targeted to this specific animal group) could be recommended. In addition, the implementation of risk based surveillance, would certainly improve the efficiency of the use of resources.

In areas where wildlife constitutes a threat to FMDV transmission, building fences at the fringes of game reserves to avoid contact between wild and domesticated animals has been adopted in some regions as a FMD prevention method. Also, given the fact that human activities through several pathways could be an important risk factor, the enhancement of compliance of biosecurity measures and the awareness of all stakeholders (e.g. farmers and veterinarians) should be taken into consideration in planning control options.

In some FMD endemic countries, the World Organisation for Animal Health (OIE) has recognized zones within the country (such as Botswana) that are allowed to export livestock on the international market. For these areas, it is highly desirable to understand and model the risks of FMD importation in FMD free zones. This assessment could thereby assist decision-makers during further outbreaks by implementing appropriate measures in due time. Consequently, the application of modelling including epidemic models could be warranted, even in an endemic setting. A valuable modelling study, recently carried out in an endemic country is illustrative and strongly encouraging for the application of models especially in areas where the threat of disease is persistent. Indeed, by catalytic and reverse catalytic models applied to serological data to estimate the force of infection and the rate of waning immunity and to detect periods of sustained transmission, Pomeroy *et al*.,[112] were able to reconstruct the historical burden of FMDV in Cameroon and to quantify control efforts necessary to stop the transmission. Additionally, in recent years, relevant studies demonstrated the feasibility of implementing epidemiological modelling based on simulations in endemic areas in SSA [113] as well as in countries where FMD free zones exist, such as in southern Africa [114–117]. Dynamic models such as SEIR are widely used and have the advantage of simplicity, but some care is needed in their application to FMDV infection, especially in endemic settings. For instance, one of the problems is that the compartments susceptible, exposed, infectious and recovered correspond only imperfectly to the states that can be defined in the field as FMDV can be demonstrated by the detection of virus, the detection of antibodies or by the appearance of clinical signs [118–119]. There is, however, no simple correspondence between these assays and whether or not the host is exposed, infectious or recovered as for example animal may become infectious before the clinical signs appear [120–121]. Consequently, from our point of view, the catalytic and reverse catalytic models developed by Pomeroy *et al*., [112] and applied in FMDV infectious in endemic situation would be interesting for future research on FMD modelling in Africa where the disease remain a serious threat for livestock development.

## Conclusions

In conclusion, the findings of this systematic review reveal that apart from models that were developed following the 2001 FMD outbreak in the United Kingdom, there are a number of publications describing modelling studies carried out in countries where FMD is endemic. Additionally, this review pointed out the unavoidable prerequisites of good-quality data to perform modelling studies with the ultimate goal to understand the epidemiology, to plan and to evaluate control programs of FMD even in countries where the disease is endemic. Certainly, FMD could be effectively controlled, if certain conditions are met. The recommended measures to be adopted include a regional approach to disease control and setting up global or regional surveillance partnerships. In addition, political and administrative authorities should consent more resources to strengthen veterinary services and the veterinary laboratory capacities especially in developing countries where FMD is endemic. When these steps are achieved, improving the data collection and the disease reporting system combined with appropriate analysis and implementation of appropriate interventions could possibly have a positive impact on FMD management and control at either the regional or national level.

## Supporting information

S1 Table PRISMA check list. Legend: Adapted from: Moher D, Liberati A, Tetzlaff J, Altman DG, The PRISMA Group (2009). Preferred Reporting Items for Systematic Reviews and Meta-Analyses: The PRISMA Statement. PLoS Med 6(6): e1000097. doi:10.1371/journal.pmed1000097. For more information, visit: www.prisma-statement.org.
(PDF)

S2 Table Search strategies and results for PubMed & Scopus databases. (PDF)

S3 Table Description of the included studies in the systematic review. Legend: Two articles [18] and [87] related to qualitative risk assessment were not included in this table. In the first paper [18], the authors have highlighted the importance of the risk analysis based on which policy changes has been implemented to control the epidemic that occurred in UK in 2001. In the second article [87], the authors described a risk assessment conducted with local expert’s opinions. They concluded that FMDV entry risk pathways in Mongolia were estimated high in relation with livestock movements.
(DOCX)
