# Supplementary material for: Review of epidemiological risk models for foot-and-mouth disease: Implications for prevention strategies with a focus on Africa
Source: PLoS One. 2018 Dec 13;13(12):e0208296. doi: 10.1371/journal.pone.0208296 (PMC6292601; doi:10.1371/journal.pone.0208296)
Supplement: S2 Table — (PDF) [file pone.0208296.s002.pdf]

S2 Table. Search strategies and results for PubMed & Scopus databases

Last date of search	Database consulted	Search algorithms applied	Results
15-12-16	Pubmed<br>Scopus	Foot-and-Mouth Disease AND Epidemiology	1170<br>790
Subtotal 1			*1960*
15-12-16	Pubmed<br>Scopus	Foot-and-Mouth Disease AND Epidemiology AND Risk assessment*	56<br>37
Subtotal 2			*93*
15-12-16	Pubmed<br>Scopus	Foot-and-Mouth Disease AND Model*	466<br>223
Subtotal 3			*689*
15-12-16	Pubmed<br>Scopus	Foot-and-Mouth Disease AND Risk factor AND Model*	95<br>84
Subtotal 4			*179*
15-12-16	Pubmed<br>Scopus	Foot-and-Mouth Disease AND Quantitative AND Risk AND Assessment	20<br>36
Subtotal 5			*56*
15-12-16	Pubmed<br>Scopus	Foot-and-Mouth Disease AND Qualitative AND Risk Assessment	8<br>10
Subtotal 6			*18*
15-12-16	Pubmed<br>Scopus	Foot-and-Mouth Disease AND Spread AND Model*	154<br>171
Subtotal 7			*325*
15-12-16	Pubmed<br>Scopus	Foot-and-Mouth Disease AND Transmission AND Model*	194<br>204
Subtotal 8			*398*
**Total of records**			**3718**
