# Supplementary material for: Review of epidemiological risk models for foot-and-mouth disease: Implications for prevention strategies with a focus on Africa
Source: PLoS One. 2018 Dec 13;13(12):e0208296. doi: 10.1371/journal.pone.0208296 (PMC6292601; doi:10.1371/journal.pone.0208296)
Supplement: S3 Table — Legend: Two articles [18] and [87] related to qualitative risk assessment were not included in this table. In the first paper [18], the authors have highlighted the importance of the risk analysis based on which policy changes has been implemented to control the epidemic that occurred in UK in 2001. In the second article [87], the authors described a risk assessment conducted with local expert’s opinions. They concluded that FMDV entry risk pathways in Mongolia were estimated high in relation with livestock movements. (DOCX) [file pone.0208296.s003.docx]

**S3 Table. Description of the included studies in the systematic review**

1. ***Modelling FMD risk factors and spatiotemporal analysis***

| **Ref.** | **Authors and year** | **Country of interest** | **Study purpose** | **Nature of data** | **Model used** | **Identified main risk factors /implications** | **Recommendations on FMD control** | **Limitations of the study** |
| --- | --- | --- | --- | --- | --- | --- | --- | --- |
| [42] | Allepuz *et al*., 2015 | Tanzania | Identification of factors associated with the spatiotemporal distribution of FMD | Retrospective data | Bayesian mixed-effects spatial | Animal movement Human activity via communication networks (roads) Transboundary movements Contact with wildlife | Prohibition or restriction of animal movements during FMD outbreaks Establishment of fences between wild and domesticated animals around reserve game | No data about outbreaks related to small ruminants and pigs. Lack of data on serotypes in the database |
| [41] | Alkhamis *et al.*, 2009 | Israel and Palestine | Identification of spatiotemporal distribution and direction of spread of clusters of reported FMD outbreaks | Retrospective data | Space-time permutation | Detected clusters suggested a seasonal pattern of occurrence of FMD outbreaks Season at high risk: spring (from April through June) | Selective vaccination campaigns, surveillance activities and control of movements before and during season at higher risk | A relative small geographical extension of the clusters was detected by the model applied. This study highlighted the imperfect sensitivity of the surveillance system due to limited ability of the veterinary services to detect and report outbreaks of the disease. |
| [43] | Ayebazibwe *et al*., 2010 | Uganda | Elucidation of possible patterns, risk factors and characteristics of occurrence of outbreaks of FMD | Retrospective data and questionnaire | Analysis of variance (Kruskal–Wallis rank test) and Logistic regression | Seasonal pattern observed for the occurrence of FMD outbreaks. Season with higher frequency: Months with lower rainfall (dry season) Main risk factor: Livestock movements (for searching pasture and water) | Consideration of the season pattern of the disease and animal movements for planning FMD control strategies in the country | Lack of geo-reference data and non-adherence to standardised outbreak reporting formats and schedules |
| [44] | Bessell *et al*., 2010 | England | Risk factors of susceptibility of holdings to FMD during the 2001 epidemic in Great Britain following the imposition of a national ban on the movements of susceptible animals. | Retrospective data | Logistic-regression | Shorter distances to the nearest infectious source High densities of cattle and sheep | Biosecurity and animal movement restriction | One of the limitations of using the GLMM framework was that the numbers of animals on the holding could not be included as a predictor due to their zero-inflated distribution. |
| [45] | Bessell *et al*., 2010 | England | Determination of whether FMD infectiousness is homogeneous with all farms equally likely to transmit infection, or whether there is significant variation in infectiousness between farms. | Retrospective data | Logistic-regression | High densities of cattle and sheep |  |  |
| [56] | Branscum *et al.*, 2008 | Turkey | Understanding and quantification of FMD occurrence (Spatiotemporal distribution) | Retrospective data | Bayesian spatiotemporal regression | High animal density |  |  |
| [88] | Bronsvoort *et al*., 2004 | Cameroon | Quantification of herd-level risk factors for herdsman-reported FMD | Sample of herds selected and questionnaire | Logistic-regression | Buying in cattle from markets Mixing of herds at watering points Going on transhumance Close contact between buffalo and herds located near the administrative division | Vaccination before going on transhumance could effectively reduce the incidence of the disease as because of the livestock production system, restricting contacts between herds either on transhumance or at drinking places would be almost impossible. | In this study, only cattle are taken into account, excluding other FMD susceptible species such as small ruminants and possibly pigs. Some variables of the questionnaire were subject to confusion with other Like “going on transhumance in the last year” with “ever seeing buffalo near the herd”. |
| [46] | Chhetri *et al.*, 2010 | Nepal | Quantification of the relation between hypothesized epidemiological factors and determination of the spatial distribution of FMD. | Retrospective data and a questionnaire | Spatial scan and Bayesian mixed effects Poisson regression | FMD risk was spatially clustered in specific areas of the country Higher risk areas were associated with the size of human and buffalo populations and with the number of veterinary technicians. | In addition to some recommendations made above in this case as elsewhere it is important to veterinary technicians to strongly follow biosecurity measures (if any) during their intervention. | As noted by the authors, this study alone could not clearly explain the reasons for these risk factors, this is probably due to the lack of strong reliability and accuracy of recorded data. |
| [61] | Dean *et al*., 2013 | Togo/West Africa | Assessment of the potential risk of regional disease spread through trade routes | Questionnaire | Stochastic simulations | The market network of northern Togo is a potential factor for disease spread including FMD between West African countries. | Implementation of regional approach to animal disease surveillance, prevention and control. | Linguistic complexity of the study zone, data errors due to incorrect interpretation are possible. Data only capture cattle trade through the formal market system. Given that informal trade also occurs, the scale of cross-border cattle trade is likely to have been underestimated. Furthermore, small ruminant cross-border trade has not been considered in this study. |
| [61] | Dukpa *et al*., 2011 | Bhutan | Quantification of the herd-level factors associated with the occurrence of FMD in sedentary herds | Questionnaire | Logistic regression | Mixing of cattle at grazing areas | Infected animals should be kept in quarantine before their reintroduction in the herd | Recall bias, interviewer bias, and failure to validate the responses to the questionnaire by repeating the questionnaire among the same respondents |
| [57] | Ellis-Iversen *et al.*, 2011 | England | Identification of risk factors for prioritizing FMD surveillance and control schemes | Case control study using Questionnaire | Logistic regression | Farms with less biosecurity had a higher risk of FMD infection during the outbreak | To enhance compliance of biosecurity measure | Higher environmental risk scores for farms increased the odds of becoming infected by three, although the statistical evidence for this association was weaker. The number of movements into the livestock areas did not appear to be associated with FMD status which contrasts with findings of many studies of risk factors for FMD |
| [58] | Elnekave *et al.*, 2015 | Israel | Evaluation of the risk factors for FMD in extensive production systems of grazing beef herds | Case control study using Questionnaire | Logistic regression | Presence of young calves under 6 months of age (high susceptibility) increase FMDV infection  Long period between vaccination of adult cattle | To increase the frequency of vaccination among herds and to intensify surveillance where young calves are abundant | There was a relatively small sample size which probably did not allow cluster detection |
| [65] | Emami *et al.*, 2015 | Iran | Assessment of putative risk factors for FMD infection and Estimation of the prevalence of antibodies to non-structural proteins in young cattle | Serological study (cross sectional study) and questionnaire | Logistic regression | High susceptibility of young calves FMD infection was strongly associated with animal trade | Control measures should be focussed at the level of epi-unit, rather than at the level of individual animals. Furthermore, this study highlighted the need for continuous vaccine quality control. | The data did not provide evidence that older calves (likely vaccinated) were more likely to test Low Positive than younger (likely non-vaccinated) calves. Indeed, the status of calves could only be inferred based on epi-unit information about dates of vaccination rather than the vaccination status itself. |
| [59] | Fasina *et al.*, 2013 | Nigeria | Assessment of exposure factors associated with a seropositive diagnosis of FMD in cattle herds and identification of circulating FMDV strains. | Case control study using Questionnaire | Logistic regression | Mixing of herds at watering points | In endemic area, vaccination alone would not be sufficient for FMD control and should be as far as possible and depending on the husbandry system, associated with quarantine measure or restriction of movement of animals. | One of the limitation of this study was observation (exposure) bias. The accuracy of a herdsman to classify his/her cattle herd as vaccinated was not assessed in this study. |
| [47] | Gilbert *et al*., 2005 | Turkey | Analysis of spatiotemporal patterns of FMD occurrence and Exploration of factors associated with FMDV (types A, O and Asia 1) persistence and spread | Retrospective data | Logistic regression and stochastic spatial model (Meta-population approach) | The pattern of persistence differed according to serotype groups. FMDV type O was the most widespread serotype in the early 1990s in Turkey. A higher affinity of FMD type O towards sheep could explain its higher persistence. FMD occurrence was associated with host abundance, short distance contagion from adjacent provinces, and meat production-demand discrepancies (leading to live animal transport over long distances) | To perform an intensive surveillance at the identified persistence islands sites identified for an effective control plan | This study is one of the few studies to use more than one model to assess the FMD risk factors. In addition, small ruminants are also taken into account in this study. |
| [48] | Gonzales *et al.*, 2014 | Bolivia | Analysis of the influence of some variables (age, sex, production type) on either the probability that an infected animal showed clinical signs or the probability of becoming seropositive (As a supplementary material of the article). | Retrospective data | Logistic regression | Young cattle (between 12 and 24 months old) were identified as at higher risk of becoming infected. Home-bred animals had higher risk of both showing clinical signs and becoming seropositive than introduced animals from outside the colony. | Surveillance targeted to this specific animal group will probably improve the successful of vaccination campaign | This study is not specifically devoted to identify and analyze foot and mouth disease risk factors but rather to assess the performance of clinical and serological diagnosis |
| [49] | Hamoonga *et al*., 2014 | Zambia | Description of the spatial distribution of FMD outbreaks and quantification of the association between geographical features of the landscape (proximity to roads, national parks, wetland areas) and the spatial distribution | Retrospective data | Poisson point process | Distance to the nearest major international border crossing Distance to the nearest major road Wetness index and elevation.  Aggregation of cattle around communal drinking pools in drier areas of the country | In such endemic country as Zambia with limited resources, selective vaccination campaigns, surveillance activities and control of movement, mainly targeted to high risk areas should be performed for an efficient use of resources | Although, the point process modelling approach presented in this study provided an effective means for performing such risk factor analysis, some variables could lead a confusion to each other such as the proximity to railways which was confounded by one or more of the other explanatory variables included in the point process model. |
| [50] | Hayama *et al.*, 2012 | Japan | Analysis of risk factors in local spread by selecting local clusters and clarification of factors associated with local spread at the within-cluster level. | Retrospective data | Logistic regression | Pig farms had a greater risk of inducing local spread than did cattle farms Larger cattle farms were more highly affected by local spread than were small cattle farms or pig farms. | Taking into account these findings, if resources are available, pre-emptive culling and emergency vaccination should be implemented to promptly control the spread of FMD | This study did not consider secondary infections between affected farms in the local spread. The consequence of this simplification is probably the overlook of the detailed mechanism of local spread. |
| [51] | Jemberu *et al*.,2015 | Ethiopia | Determination of the incidence, distribution, risk factors, and serotypes of FMD outbreaks in Ethiopia | Retrospective data and questionnaire | Linear regression  Logistic regression | Production system (market oriented system versus subsistence systems)  Presence of a major livestock market and/or route Adjacency to a national park or wild life sanctuary Sheep and goat densities | Given the complexity of the epidemiology of FMD in this country, several control measures must be planned and implemented: Vaccination associated with a restriction of movement of animals in the outbreak area,  Setting compulsory quarantine at the cattle market,  Epidemiological surveillance involving both small ruminants, pigs and wildlife | The study has a possible reporting bias due to the use of a questionnaire data rather than a prospective collection of objective data. In addition, the study has a low geographical resolution because of use of districts as sampling units, which themselves are not strictly uniform entities |
| [52] | McLaws *et al.*, 2009 | United Kingdom | To identify factors associated with the early detection of FMD-infected premises during the 2001 epidemic in the UK. | Retrospective data | Logistic regression | Lack of early screening/detection | Reporting by farmers and initiatives that increase farmer education and awareness should be encouraged | There was evidence of over dispersion in the final model, because the deviance chi-squared value was greater than its degrees of freedom. This might be an indication that the model had not accounted for clustering in the data, or that an important variable was not included in the model |
| [62] | Megersa *et al.*, 2009 | Ethiopia | Investigation of the potential risk factors associated with FMD occurrence. | Cross-sectional study (seroprevalence study) | Logistic regression | Production system (pastoral) | The same control measures formulated above can be applied in this context | The analysis of risk factors was performed in this study on the basis of a seroprevalence study. The limitation of this study could be related to the imperfection of the sensitivity and specificity of the serological test applied. Moreover, the role of wildlife has not been investigated in this study. |
| [60] | Muroga *et al*., 2013 | Japan | Examination of risk factors associated with FMD transmission between farms | Case control study using Questionnaire | Logistic regression | Movement of people and vehicles | To enhance compliance of biosecurity measure | As noticed by the authors of this study, it exists a possible bias due to the case–control study approach based on questionnaires. In fact, while case–control studies can identify relationships between possible risk factors and the occurrence of disease, it provides no information about cause and effect. |
| [36] | Perez *et al.*, 2005 | Iran | Identification of high-risk areas of FMD | Retrospective data | Spatial scan statistic and multivariate regression | Significant clusters of FMD were identified that coincided with roads, neighbouring countries, and high-density population areas | An integrated control approach at regional or continental level is strongly required | In this study, the lack of information on the number of animals or herds distributed in time and space could be a serious handicap to the clusters analysis. Furthermore, this study would not have a precise investigation of risk factors related to disease transmission |
| [39] | Perez *et al.*, 2006 | Pakistan | Estimation of the spatial risk of FMD in Pakistan | Retrospective data | co-kriging | International animal-trade route | Same recommendation as above | The findings of this study are formulated from a model that is based on a probability interpolation method which does not take into account the variability (deterministic) |
| [53] | Picado et al., 2011 | Tanzania | Investigation of the the spatiotemporal pattern of transmission | Retrospective data | K-Function and Space–time permutation | High concentration of wildlife, extensive livestock movement and the pastoral mode of livestock farming | See recommendation for reference 33 | These models only take into account the FMD-affected villages and the area delimited by them each year. |
| [37] | Picado et al., 2007 | United Kingdom | Description of the temporal and spatiotemporal patterns of disease occurrence | Retrospective data | space–time K-function | Shorter distances to the nearest infectious source | See recommendation for references 44 and 57 | The limited data required for its implementation would make this technique easy to incorporate into the real-time management of epidemics. However, this simplicity is also one of its limitations, as the role of farm characteristics in infectiousness in time and space is ignored. |
| [54] | Sinkala *et al*., 2014 | Zambia | Investigation of the spatial distribution of FMD and identification of significant FMD clusters | Retrospective data | Space–time permutation | Existence of statistically significant FMD in agreement with areas known to be at high risk of FMD in Zambia. The main supposed risk factors are:  Livestock movement Livestock marketing (movement driven by incentives related to differences in price of livestock) Presence of African buffalo | In addition to other control options mentioned above including those for endemic countries, a periodically risk-based surveillance and further investigations into risk determinants within the identified clusters should be performed. | The findings are limited to the data set investigated and do not factor in the level of under-reporting, unconfirmed samples, livestock or wildlife densities and serotype variations. |
| [55] | Volkova *et al*., 2011 | Scotland | pattern of transmission. | Retrospective data | Logistic regression | No direct movements of livestock identified by contact tracing. Twelve holdings in Scotland were identified as being at risk of FMD due to potential indirect contacts. Probability for a single FMD-infected livestock holding in Scotland remaining undetected dropped from 100 per cent to lower than 50 per cent during two weeks under all of the scenarios of incubation periods. | The restriction of animal movements in local markets (for free FMD countries) should be based on consistent arguments. Risk modelling with reliable data could thus assist in decision making. | Unfortunately, it often lacks reliable data to implement these types of studies |
| [64] | Vosloo *et al.*, 2009 | South Africa | Investigation of the dynamics of FMD infection in three geographically distinct impala populations in selected ecologically diverse habitats of the KNP in order to determine the role of these impala in the FMD maintenance | Seroprevalence and census data | Logistic regression | Summer and autumn have been identified as highest risk periods associated with seroprevalence contrasting with data derived from clinical outbreaks that indicated that most outbreaks in impala occur at the end of the dry season.  Author’s assumptions: the progression of infection within the herd over time after the initial dry season outbreak, hence, animals infected during the dry season and sampled within the next 6–9 months (wet season) are more likely to be seropositive.  Impala in the presence of infected buffalo herds should be considered as a risk factor for the spread of FMD to other susceptible species in southern Africa | Strategies for FMD control through vaccination should include consideration of the season. It is also important to establish fences between wild animals and domestic animals around reserve | This study demonstrates the difficulty of using serology to estimate the prevalence of FMD infection in wildlife even without vaccination that could further influence the interpretation of data. |
| [38] | Wilesmith *et al.*, 2003 | United Kingdom | Assessment of the spatiotemporal interaction of FMD infection risk and description of the spatial and temporal features of the incidence of FMD epidemic in 2001 in UK | retrospective data | Spatiotemporal (space-time K-function) | Many cattle holdings infected early in the epidemic (creating a high environmental viral load) Relatively large amount of medium-to-long-distance spread of the virus associated with seasonal farming activities-compounded to some extent by the movement of people and vehicles between disaggregated farm land parcels.  The interaction of disease risk in this area showed that premises remained infectious for longer throughout at least three months (May, June and July), consistent with delays in disease detection during this period. | As for other free FMD countries, recommendations are primarily in the preventive sense including risk modelling activities aiming to determine appropriate control measures in case of outbreaks | The methodology used consider time and space independently, hence the ability to describe the risk of infection attributable to spatio–temporal interaction provides further insight: (1) identifying the extent of ‘contagiousness’ in space (and therefore providing an objective means for defining suitable pre-emptive culling distances) and (2) in time (indicating how quickly infection risk is ‘extinguished’ after a holding becomes infected) |

1. ***Quantitative risk assessment model for FMD***

| **Ref.** | **Authors and year** | **Country of interest** | **Estimated risk** | **Type of Model used** | **Key variables described/included in the model** | **Risk probability/Key findings** | **Sensitivity analysis** | **Risk categorization** |
| --- | --- | --- | --- | --- | --- | --- | --- | --- |
| [73] | Adkin *et al.*, 2008 | United Kingdom | Frequency with which meat waste from ships or aircraft might expose British livestock to infection with FMD | Stochastic | Aircraft cabin waste, Aircraft galley waste, Cruise waste, Container waste | Total weight of FMD contaminated waste estimated to be 26 kg per year. Mean value of Frequency of livestock infection in GB = 0·0007 per year (1429 years between outbreaks of FMD due to ship and aircraft waste) | These estimates were affected by three principal uncertainties: (1) the prevalence of FMD; (2) the probability of the waste being removed and fed to pigs; and (3) the probability that overboard dumping of contaminated waste might expose livestock to FMD | Low |
| [76] | Asseged *et al.*, 2012 | United States | Assessment of the probability of introduction of FMDV into the USA via the importation of cloned bovine embryos | Stochastic | risk associated with somatic cell donor, risk associated with oocyte donors, risk associated with transferable embryo | Mean Probability of introducing FMDV via cloned embryos was estimated to be 3.1 × 10^-7^ | The inputs showing the highest variability/uncertainty were: the FMDV status of the cumulus–oocyte complex, the effectiveness of the embryo washing protocol and embryo testing procedures, and the FMDV status of oocyte donors. | Very low |
| [78] | Carpenter et al., 2007 | United States | Estimation of the potential spread of FMD if infected livestock had been exhibited at the 2005 California State Fair | Stochastic | Livestock species (dairy cattle, dairy goats, pygmy goats, mixed), number of exhibitors, number of exhibitors surveyed and number of respondents returning animal to commercial premises | The mean probability that at least 1 animal that became infected with FMD would subsequently leave the state ranged from 28% to 96% as the number of index cases increased from 1 to 10, respectively. | NA | High |
| [67] | de Vos et al., 2010 | Netherlands | Analysis of the risk of exporting FMD-infected pig carcasses from a vaccinated area | Stochastic | Herd prevalence, within-herd prevalence and probability of detection at slaughter | The probability that a processed carcass was derived from an FMD-infected pig (Pcarc) was on average 2.0 × 10−5 directly after final screening, and 1.7 × 10−5 after a six-month | The sensitivity analysis indicated that P_carc_ was strongly influenced by the number of herds in the vaccinated area. | Very low |
| [68] | Hartnett *et al*., 2007 | United Kingdom | Estimation of the annual frequency with which the illegal importation of meat will result in infection with FMD in the GB livestock population and investigation of the contribution of a number of factors to the final estimates of risk. | Stochastic | Illegal import, illegal personal, illegal commercial and sized meat | The total amount of illegal meat entering GB each year is estimated on average to be 11,875 tonnes. This volume of illegal meat is made up of imports from the 14 regions considered in the model. The results indicate that approximately 95% of the estimated risk to susceptible animals from illegal meat is associated with illegal meat arriving in personal baggage. | The scale factors are highly influential in the risk estimation process. Underestimation of the flow from any give region may result in an underestimation in the risk estimate. | High |
| [77] | Jones *et al*., 2004 | United Kingdom | Assessment of the risk of new outbreaks occurring as a result of the six burning pyres during FMD epidemic in 2001 in UK. Estimation of the distance-dependent probabilities of infection of either an animal or a holding from exposure to the virus within each of the plumes. | Stochastic | Distance from holding to pyre, Estimated level of exposure, Probability of infection given exposure and the expected number of outbreaks | The probabilities of a cow or sheep being infected were estimated, with 95 per cent certainty, to be less than 0.003 and 0 0004, respectively, with the highest probability for animals on a holding within a kilometre of a pyre. For the two premises which became infected under the plumes from pyres 1 and 5, the estimated risk of infection was less than that for equally susceptible farms which were closer to the pyre but did not become infected. | Some of the uncertainty in the probabilities estimated from the model results from the uncertainty in the dose response parameter. | Low |
| [74] | Lin *et al.*, 2009 | Taiwan | Prediction of FMD entrance caused by passengers who illegally carry meat products of cloven-hoofed animals through international airports into a country. | Stochastic model: Data used for analysis were records of illegal meat product carriers from areas A and B intercepted at an international airport in Taiwan. | Prevalence of FMD, commodity factor event (the contaminating virus in meat products that persists throughout processing of products), transportation, storage, and distribution (TSD) factor event (the survival of the virus in meat products after processing through international transportation) the passenger event, which represents no intercepted passengers who illegally carry meat products contaminated with the FMD virus | The probability of the passenger event was hypothesized with the odds of intercepted passengers (A and B) and estimated via logistic regression. The results showed that the odds of passengers being intercepted by beagles were higher than those intercepted by Customs. The probability of FMD virus risk caused by FMD factors from area A was 149 times lower than that from area B. The probability of FMD virus risk caused by the passenger event from area A (3.11 × 10−10) was four times lower than the corresponding probability from area B (2.00 × 10−7). | For sensitivity analysis, beta distribution was used to describe the value of period prevalence, and the distribution of the duration of viremia. Since the cases and animal populations were reported voluntarily and no other informative reports were accessible, the calculated prevalence is thus imprecise and may be underestimated. | Very low |
| [70] | Martinez-Lopez *et al*., 2008 | Spain | Estimation of the probability of an FMD epidemic occurring in Spain as a consequence of the introduction of live animals into the country from another EU member country. | Stochastic | Number of animals imported, Country-specific probability of infection, Probability of survival to FMD-infection, Probability of exporting into Spain, Probability of entering into an agricultural operation, Probability of establishing an effective contact | The mean probability of FMDV introduction into Spain via import of live animals per year was estimated as 2.36 × 10−2, with a 95% PI of (7.37 × 10−6, 1.61 × 10−1), which corresponds to approximately one outbreak every 40 years. The probability of FMDV introduction into Spain was estimated to be more likely to occur via importation of live pigs than through importation of other species susceptible to FMDV infection. | The low correlation between the probability of FMDV introduction into Spain and the number and density of susceptible species in the province (Rs < 0.6) suggests that knowledge of the value of these parameters alone is not sufficient to predict the risk of FMDV introduction into Spain | Low |
| [79] | Martinez-Lopez *et al*., 2014 | Peru | Assessment of the risk for potential FMD re-introduction into Peru and to quantify the FMD spread and economic impact associated with hypothetic FMD epidemics. | Spatial-stochastic | Distribution and abundance of susceptible population, structure and frequency of between-farm animal movements as a proxy for potential disease introduction or spread, within- and between-farm FMD-transmission, associated direct and indirect costs | The mean (95% probability interval) number of outbreaks, infected animals, epidemic duration, and direct costs were 37 (1, 1164), 2152 (1, 13, 250), 63 days (0, 442), and US$ 1.2 million (1072, 9.5 million), respectively. | Changes in the parameters related with the probability of local spread, the latent period and the probability of farm detection based on clinical signs were the ones that mostly influenced model results. Therefore, the reduction of the uncertainty in those parameters will increase model reliability. | High |
| [71] | Miller *et al.*, 2012 | USA | Estimation of the probability of an outbreak of FMD occurring in the USA as a result of the importation of livestock and to understand the sensitivity of the results to the various risk parameters used in estimating the probability. | Stochastic | Number of livestock of species s imported from country c to the USA, Probability of having an undetected case of FMD in country c when animals are shipped to the USA during the period of time beta (one year), Number of animals in country c expected to be infected before the detection of the epidemic, Population of susceptible animals in country c, Total number of herds in country c, Probability of surviving the trip, Probability of establishing an effective contact and Probability of detecting FMD in quarantine stations in the USA before livestock enter domestic premises. | The total probability of introduction into the USA of FMD from imported livestock is estimated to be 0.415% per year, which is equivalent to one introduction every 241 years. | The overall probability of FMD introduction changed only by 0.01% as herd size varied from 20 to 5,000. Sensitivity analysis showed total probability exposure changed minimally as herd size was varied | Very low |
| [8] | Schijven *et al.*, 2005 | Netherlands | Assessment of the probability of infecting dairy cows that were drinking FMDV contaminated surface water due to illegal discharges of contaminated milk. | Exponential dose-response model and beta-Poisson model | Illegal discharge of contaminated milk into sewerage, Transport to a sewage treatment plant, Discharge of biologically treated and raw sewage into surface water, Dilution of discharged sewage in surface water depending on size of STP and receiving surface water, Exposure of cows to FMD virus by consumption of contaminated surface water and probability of infection using dose-response relationship data. | The probability of infection of a herd of 53 cows in the case of a dilution factor of 44 is 8.5 × 10−5. Through this study, it was found that illegal and uncontrolled discharges of contaminated milk into the sewerage system may lead to high to very high risks to other cattle farms at 6–50 km distance of the location of discharge within 1 day. The probabilities of infections due to drinking from FMD virus contaminated large river waters are much lower than from small river waters due to more dilution. However, in the larger rivers, contamination will stretch over larger distances. | The authors made use of ranges (rather expert opinion), like for the virus concentration in milk, the difference between treated and raw discharges of wastewater, and the dilution into surface water. These ranges reflected the uncertainty about the values of these parameters. Given this range of parameter values a strong indication could be given on the significance of spreading FMDV via this pathway | Low |
| [75] | Sutmoller *et al.*, 2000 | Zimbabwe | Evaluation of the effectiveness of the containment of FMD in buffaloes within the conservancies | Stochastic | Five different scenario pathways were considered by which FMD virus could be transmitted from infectious buffalo calves within the conservancies to livestock. The following input parameters were used: size of the buffalo herd, the fraction of the herd consisting of 4–12 months-old calves that | Of the five scenarios considered, the greatest annual risk (2×10^−4^) for cattle would be from antelope jumping over the outer perimeter fence of the conservancy and infecting cattle on the outside. The other transmission scenarios (including air-borne transmission) had a FMD risk that was low to very low. | NA | Low |
| [72] | Wongsathapornchai *et al.*, 2008 | Malaysia | Assessment of the likelihood of an introduction of FMD through terrestrial movement of livestock. | Stochastic | Movement of livestock and Probabilities of livestock having FMD and of FMD infection going undetected during import processes. The probability of an animal accepted for import having FMD was also assessed. | The simulation yielded an average consignment prevalence of 10.95%. Typically, each animal in a quarantine facility had a 2.7% chance of having an inapparent form of FMD infection; hence, it was likely an animal would not be identified as infected. Findings revealed that the mean probability of an animal accepted for import having FMD was 2.9%, and the risk was as high as 11%. | The value for P_S_ (probability of infected livestock having detectable signs during quarantine) had an effect on the degree of risk for introduction of FMD. The assumed number of animals with clinical signs of FMD increased, the probability of the introduction of FMD into the MTM zones decreased. | High |
| [69] | Wooldridge *et al.*, 2006 | United Kingdom | Risk assessments examining the disease risk to the GB livestock population of FMD, CSF, ASF and SVD from the illegal importation of any meat product from any region in the world. | Stochastic | Flow of illegally imported meat into GB, probability that meat is contaminated with a virus, specifically FMDV ant the other viruses, exposure pathways and probability and frequency of infection in GB livestock caused by contaminated meat | Mean Flow of the quantity of illegally imported meat that is contaminated with FMDV per year into Great Britain = 214.2 Kg Mean Frequency of infection per year for infection with FMDV as a result of the illegal importation of meat and meat products into Great Britain = 0.015 | NA | NA |

1. ***Qualitative risk assessment model for FMD***

| **Ref.** | **Authors and year** | **Country of interest** | **Risk assessed** | **Overall risk** |
| --- | --- | --- | --- | --- |
| [82] | Jori *et al.*, 2009 | South Africa | Risk of FMDV release outside Kruger National Park and subsequent spread in the buffer zone with vaccination | Moderate |
| [85] | Moutou *et al.*, 2001 | Russia and Europe | Probability (risk) of occurrence of FMD | Moderate |
| [83] | Paton *et al.*, 2010 | Not specified | Risk associated with International Trade in Deboned Beef | Low |
| [86] | Pharo, 2002 | New Zealand | Introduction of FMD virus into New Zealand in legally imported animals and animal products | Low |
| [84] | Sutmoller, 2001 | Not specified | Risk posed by cattle slaughtered during the carrier stage for the international beef trade | Negligible |
